# Renal nerve ultrastructural alterations in short term and long term experimental diabetes

**DOI:** 10.1186/1471-2202-15-5

**Published:** 2014-01-05

**Authors:** Karina Laurenti Sato, Luciana Sayuri Sanada, Renata da Silva Ferreira, Maria Carolina del Bem de Barros Oliveti de Marco, Jaci Airton Castania, Helio Cesar Salgado, Randy Alan Nessler, Valeria Paula Sassoli Fazan

**Affiliations:** 1 Departments of Neurosciences and Behavioral Sciences, School of Medicine of Ribeirão Preto, University of São Paulo, Ribeirão Preto, São Paulo, Brazil; 2 Departments of Physiology, School of Medicine of Ribeirão Preto, University of São Paulo, Ribeirão Preto, São Paulo, Brazil; 3 Central Microscopy Research Facility, The University of Iowa, Iowa City, IA, USA; 4 Dept. of Surgery and Anatomy, School of Medicine of Ribeirão Preto, University of São Paulo, Ribeirão Preto, São Paulo, Brazil

**Keywords:** Renal nerve, Ultrastructure, Unmyelinated fibers, Diabetic neuropathy

## Abstract

**Background:**

Despite the evidence that renal hemodynamics is impaired in experimental diabetes, associated with glomeruli structural alterations, renal nerves were not yet investigated in experimental models of diabetes and the contribution of nerve alterations to the diabetic nephropathy remains to be investigated. We aimed to determine if ultrastructural morphometric parameters of the renal nerves are affected by short term and/or long term experimental diabetes and if insulin treatment reverses these alterations. Left renal nerves were evaluated 15 days or 12 weeks (N = 10 in each group) after induction of diabetes, with a single injection of streptozotocin (STZ). Control rats (N = 10 in each group) were injected with vehicle (citrate buffer). Treated animals (N = 10 in each group) received a single subcutaneous injection of insulin on a daily basis. Arterial pressure, together with the renal nerves activity, was recorded 15 days (short-term) or 12 weeks (long-term) after STZ injection. After the recordings, the renal nerves were dissected, prepared for light and transmission electron microscopy, and fascicle and fibers morphometry were carried out with computer software.

**Results:**

The major diabetic alteration on the renal nerves was a small myelinated fibers loss since their number was smaller on chronic diabetic animals, the average morphometric parameters of the myelinated fibers were larger on chronic diabetic animals and distribution histograms of fiber diameter was significantly shifted to the right on chronic diabetic animals. These alterations began early, after 15 days of diabetes induction, associated with a severe mitochondrial damage, and were not prevented by conventional insulin treatment.

**Conclusions:**

The experimental diabetes, induced by a single intravenous injection of STZ, in adult male Wistar rats, caused small fiber loss in the renal nerves, probably due to the early mitochondrial damage. Conventional treatment with insulin was able to correct the weight gain and metabolic changes in diabetic animals, without, however, correcting and / or preventing damage to the thin fibers caused by STZ-induced diabetes. The kidney innervation is impaired in this diabetic model suggesting that alterations of the renal nerves may play a role in the development of the diabetic nephropathy.

## Background

Diabetes mellitus is associated with multi-organ complications being peripheral neuropathy the most common, occurring in up to 45–50% of diabetic patients [1]. Diabetic neuropathy affects both the somatic nervous system (especially sensitive nerves) and the autonomic nervous system. In terms of morbidity and mortality diabetic autonomic neuropathy is a very important issue, especially when it involves the autonomic cardiovascular control [2-5].

Alterations in the peripheral nervous system of genetic or experimentally induced models of diabetes have been extensively investigated. Despite that morphological alterations comparable to those that occur in the human diabetic neuropathy are not completely reproducible [6], it is expected that alterations observed in experimental models, especially in the early or acute phases, will be useful for a better understanding of the neuropathy development mechanisms [6,7].

Among other complications of diabetes, the prevalence of diabetic nephropathy is described as only 20% [1,8], compared to the high percentage of the neuropathy. Nevertheless, there is evidence that renal hemodynamics is altered in early diabetes, and this alteration would be a major contributor to the development of diabetic nephropathy [9-11]. In experimental conditions, renal enlargement is present after few days of induced diabetes [12,13], being the glomeruli of diabetic rats 30% larger in volume. This increase precedes the glomerular filtration changes [12]. Chronic alterations of the glomeruli in diabetes include enlargement of mesangial cells and matrix expansion and they correlate with the poor glomerular function in diabetic patients [14]. Despite the relatively large amount of literature on renal structure alterations either in human [14,15] or experimental diabetes [13,16,17], the morphology of the renal nerves were not yet investigated in experimental models of diabetes and the contribution of nerve ultrastructural alterations to the diabetic nephropathy remains to be investigated.

In the present study we described the ultrastructural morphology and morphometry of the renal nerves in short term and long term diabetic animals. We also show the effects of insulin treatment in the renal nerves ultrastrucutral alterations.

## Results

### Metabolic and physiological data

Body weight, blood glucose level, MAP and HR values for all experimental groups are shown in Table 1. Differences were not observed on body weight between all groups at the beginning of the experiments. Chronic controls and chronic treated animals gained weight compared to acute counterparts. Chronic diabetic animals also gained weight compared to acute animals, but the difference was not statistically significant. Acute controls were heavier that both acute diabetic groups on the final experimental day (One way ANOVA, p < 0.001; F = 28, DF = 2). For chronic groups, treated animals were heavier than controls and diabetic while controls were heavier than diabetic animals (One way ANOVA, p < 0.001; F = 73, DF = 2). As expected, blood glucose levels were significantly higher in diabetic animals, for both acute and chronic groups. Treated animals from acute group also showed high blood glucose levels compared to acute controls, while this was not evident for chronic animals. Mean arterial pressure (MAP) was significantly lower in both diabetic groups compared to controls, and also on acute diabetic group compared to acute treated group (One way ANOVA, p < 0.001; F = 10, DF = 2). Chronic controls showed higher MAP than acute controls. Heart rate (HR) was higher on acute controls compared to both diabetic groups, while chronic diabetic animals showed smaller values compared to chronic controls and treated groups (One way ANOVA, p < 0.001; F = 15, DF = 2). Also, chronic controls and chronic treated groups showed higher HR than respective acute groups. This was not observed between diabetic groups.

**Table 1:** Body weight, blood glucose level, arterial pressure and heart rate data from the six experimental groups: acute diabetic group (15 days after STZ injection), acute treated group, acute control group, chronic diabetic group (12 weeks after STZ injection), chronic treated group and chronic control group

	**Body weight (g)**	**Blood glucose (mg/dl)**	**MAP (mmHg)**	**HR (bpm)**
Acute diabetic	200 ± 4	407 ± 13	111 ± 2	250 ± 10
Acute treated	235 ± 10	218 ± 58	125 ± 1*^Ѳ^	283 ± 3
Acute control	309 ± 9*^+^	79 ± 2*^+^	121 ± 2	303 ± 3*^+^
Chronic diabetic	257 ± 22^†^	451 ± 35	124 ± 6	282 ± 11*
Chronic treated	647 ± 39^#+^	190 ± 74	138 ± 6	343 ± 14^#^
Chronic control	544 ± 17^#†Ѳ^	72 ± 3^#^	145 ± 5^#Ѳ^	348 ± 6^†^

MAP = mean arterial pressure; HR = heart rate; ^Ѳ^indicates difference compared to acute control group; *indicates difference compared to acute diabetic group; ^+^indicates difference compared to acute treated group; ^#^indicates difference compared to chronic diabetic group; ^†^indicates difference compared to chronic treated group.

### Morphological aspects

**Figure 1:**
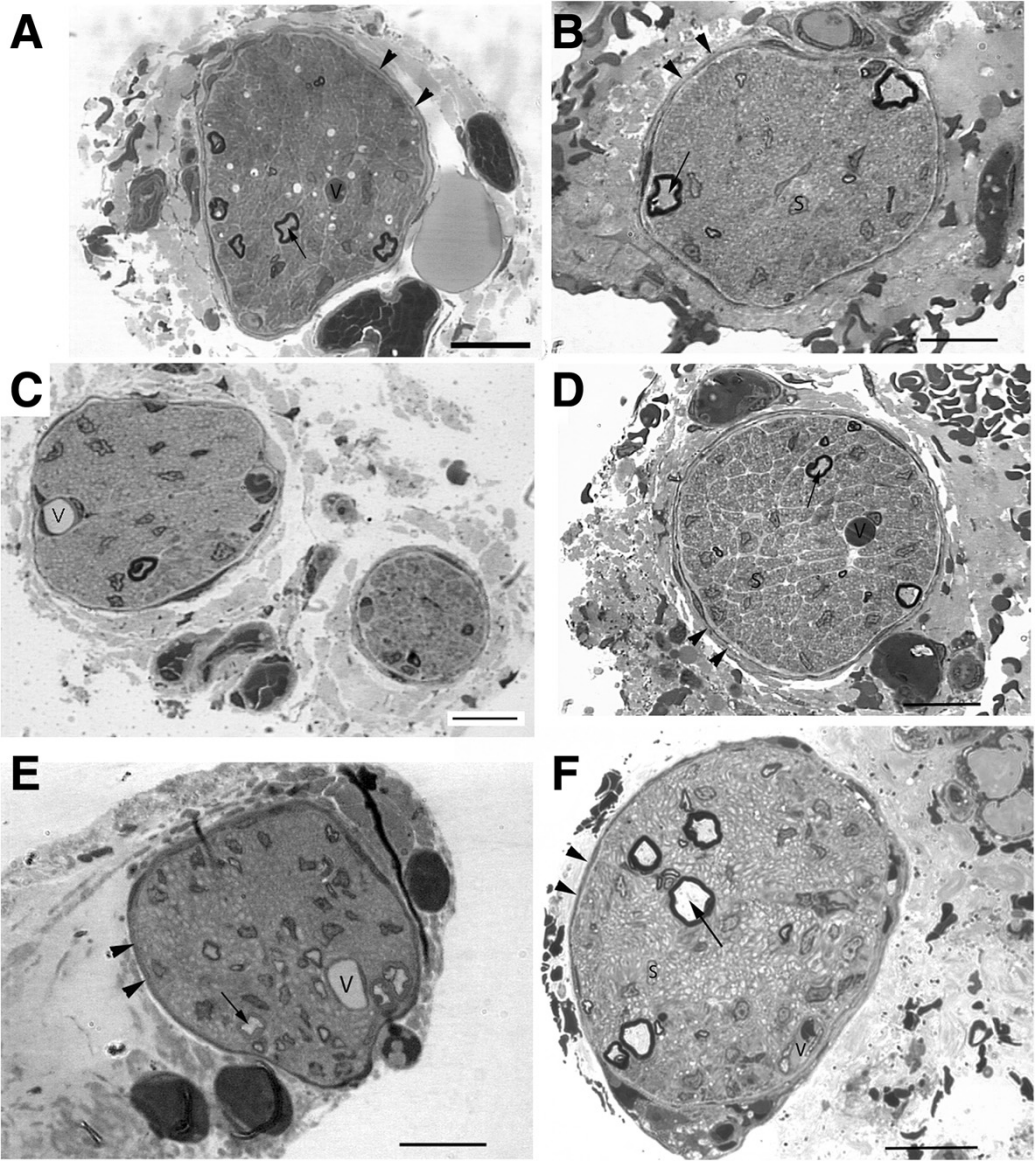
**Semithin transverse sections of renal nerves from acute control (A), chronic control (B), acute diabetic (C), chronic diabetic (D), acute diabetic treated with insulin (E) and chronic diabetic treated with insulin (F) animals.** The nerve fascicles are enveloped by a well defined perineurium (arrowheads) even when more than one fascicle is present **(C)**. Most of the endoneural space is occupied by unmyelinated fibers but few myelinated fibers (arrows) are present. No morphological differences were evident between groups at this resolution. V = capillary vessels, S = Schwann cell nucleus. Toluidine blue stained, bars = 10 μm.

**Figure 2:**
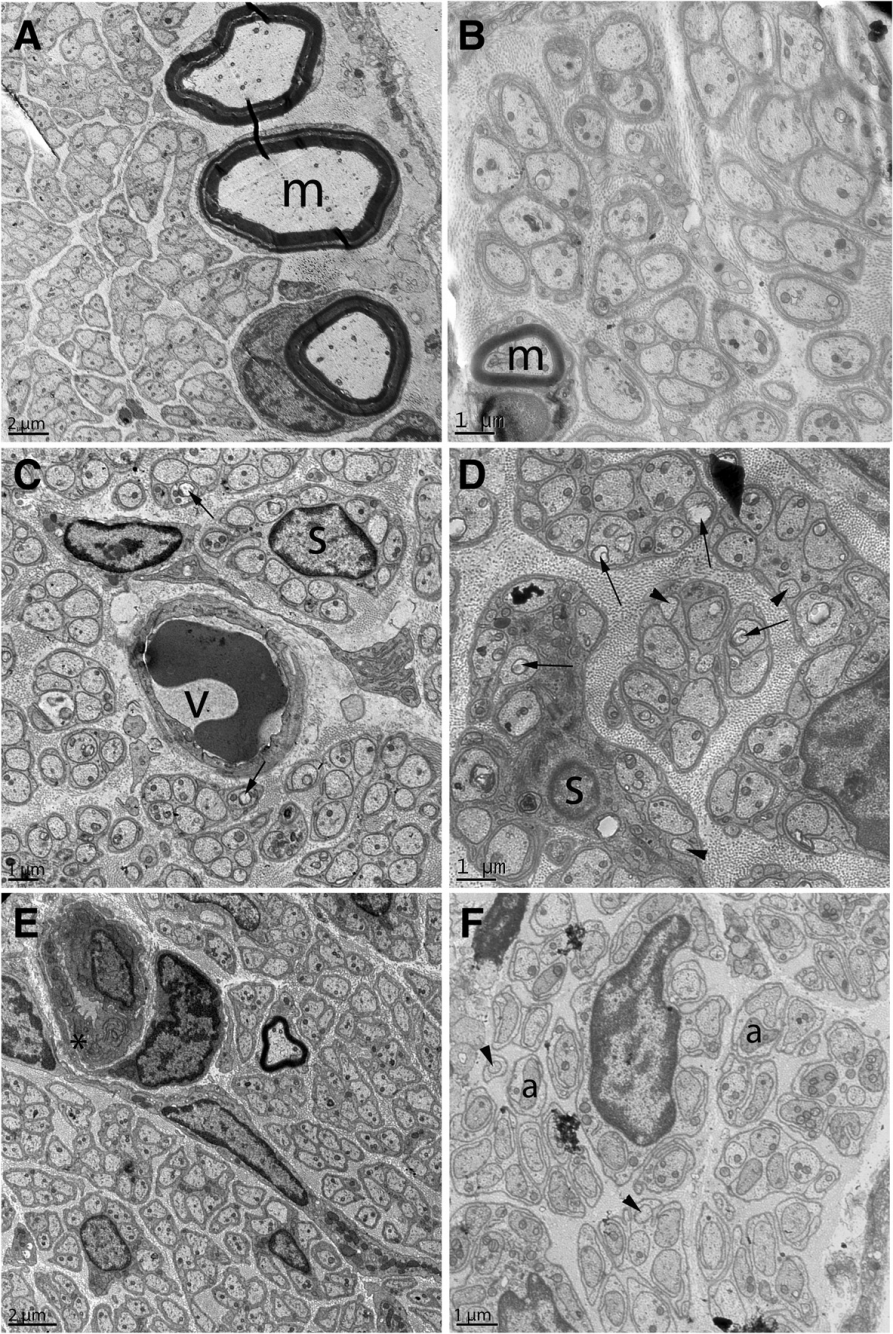
**Transmission electron microscopy cross sections of renal nerves from acute control (A), chronic control (B), acute diabetic (C), chronic diabetic (D), acute diabetic treated with insulin (E) and chronic diabetic treated with insulin (F) animals.** No ultrastructural alterations were evident on the controls that show typical Schwann cell units. Myelinated fibers are indicated by “m”. Acute diabetic nerves showed mild destruction of axonal mitochondria (arrows) and unmyelinated fiber enlargement with signs of swelling and degeneration (*). Chronic diabetic nerves presented with severe destruction of axonal mitochondria (arrows), micro-axons formation (arrowheads) and thickening of the endoneural space with an increased density of collagen fibers. Insulin treated animals showed alterations of the endoneural blood vessels such as thickening of the basement membrane (*) and lumen reduction at early stages **(E)**. In chronic stages, very small unmyelinated axons were present (arrowheads), edema of the endoneural space could be evidenced and unmyelinated fibers with atrophy (a) were frequent. Bar = 1 μm for B, C, D and F. Bar = 2 μm for A and E.

The renal nerves consisted of one up to three fascicles, each one enveloped by one or two layers of flattened cells, which constituted the perineurium (Figure 1). The endoneural space presented few large and small myelinated fibers, intermingled with a larger number of unmyelinated ones (Figures 1 and 2). Longitudinally oriented collagen fibers occupied much of the endoneural space. The larger fascicles presented one or two capillary vessels within the endoneural space. Other structures often seen in autonomic nerves, such as neuronal cell bodies or paraganglia were not observed in the renal nerves. No evident morphological differences were observed between the experimental groups in the light microscopy level. The ultrastructural qualitative analysis showed that control nerves present morphological characteristics similar to the normal renal nerves described for rodents [18,19]. Nerves from diabetic animals showed small myelinated fibers with signs of demyelination, presence of microaxons, unmyelinated fiber enlargement with signs of swelling and degeneration, Schwann cell processes not enveloping any axon, collagen pocket formation, severe mitochondrial damage and thickening of the endoneural space with an increased density of collagen fibers (Figure 2). These alteration were present in both acute and chronic groups but were more severe in the chronic diabetic animals. Treated animals’ nerves showed severe endoneural blood vessels damage, associated with basement membrane thickening of the Schwann cells and unmyelinated axon atrophy (Figure 2). Very small unmyelinated axons were also present in these groups, edema of the endoneural space could be evidenced and unmyelinated fibers with atrophy were frequent. Myelinated fibers were more preserved in the treated diabetic groups.

### Morphometric aspects

Table 2 shows the mean values for fascicular morphometric parameters, myelinated and unmyelinated fibers numbers and densities and Schwann cells number and density for all experimental groups. No differences were observed in the comparisons between the three acute experimental groups, for all parameters, except to the unmyelinated fibers density (Table 2), that showed larger values for the acute treated group compared to the acute diabetic group and acute control group (One way ANOVA, p < 0.001; F = 41, DF = 2).

**Table 2:** Average morphometric data of the renal nerves fascicles from the six experimental groups: acute diabetic group (15 days after STZ injection), acute treated group, acute control group, chronic diabetic group (12 weeks after STZ injection), chronic treated group and chronic control group

	**Acute diabetic**	**Acute treated**	**Acute control**	**Chronic diabetic**	**Chronic treated**	**Chronic control**
Fascicular area (μm^2^)	5030 ± 1200	4470 ± 619	3585 ± 672	7834 ± 1103*	6212 ± 547	5300 ± 1177
Fascicular diameter (μm)	76 ± 9	74 ± 5	64 ± 7	124 ± 19*	88 ± 4	83 ± 12
Myelinated fiber number	6 ± 2	9 ± 2	6 ± 1°	10 ± 3	13 ± 2	19 ± 7
Myelinated fiber density (number of fiber/μm^2^)	1470 ± 411	2113 ± 441	1949 ± 759	1333 ± 295°	2105 ± 292	3885 ± 1124
Schwann cell nucleus number	34 ± 4	24 ± 4	32 ± 6	44 ± 8	42 ± 3^+^	38 ± 7
Schwann cell nucleus density (number of nucleus/μm^2^)	9043 ± 1420	6013 ± 950	10650 ± 1752	5517 ± 544*	6897 ± 662	8424 ± 1606
Unmyelinated fiber number	1821 ± 330	2289 ± 342	1322 ± 230	1481 ± 175	1573 ± 218	1237 ± 98
Unmyelinated fiber density (number of fiber/μm^2^)	408219 ± 41358^+^	578601 ± 96603	379066 ± 38120^+^°	211857 ± 31431°	256855 ± 33371^+^°	288967 ± 31934

*Indicates difference compared to acute diabetic group, ^+^indicates difference compared to acute treated, °indicates difference compared to chronic control.

Comparison between the three chronic groups showed that myelinated fiber density on diabetic group is smaller than controls (One way ANOVA, p < 0.013; F = 73, DF = 2). Also, for the chronic groups, the unmyelinated fiber density is smaller on both diabetic groups, treated and untreated, compared to controls (One way ANOVA, p < 0.001; F = 52, DF = 2). No differences were observed for all other parameters investigated.

The comparison of the fascicular parameters between acute and chronic groups, for controls, diabetic and treated animals, showed larger average values on chronic groups, with values reaching statistical significance between the diabetic groups (p = 0.014). The average number of myelinated fiber was larger on all chronic groups, reaching statistical significance between control groups (p = 0.016). Average Schwann cell nucleus density was larger on both diabetic chronic groups, treated and untreated, compared to their acute counterparts, reaching statistical significance between the treated groups (p < 0,001). Also, both chronic diabetic groups showed larger average Schwann cell nucleus density compared to the chronic control group, but with no statistical significance.

Table 3 shows the mean values of the myelinated and unmyelinated fibers morphometric parameters for all experimental groups.

**Table 3:** Average morphometric data of the renal nerves’ myelinated and unmyelinated fibers, from the six experimental groups: acute diabetic group (15 days after STZ injection), acute treated group, acute control group, chronic diabetic group (12 weeks after STZ injection), chronic treated group and chronic control group

	**Acute diabetic**	**Acute treated**	**Acute control**	**Chronic diabetic**	**Chronic treated**	**Chronic control**
Myelinated fiber area (μm^2^)	14.2 ± 1.5	11.8 ± 0.8	20.8 ± 2.8°	17.8 ± 1.6°	19.6 ± 2.3°	11.1 ± 1.0
Myelinated fiber diameter (μm)	3.9 ± 0.1	3.7 ± 0.1	4.7 ± 0.3°	4.3 ± 0.2°	4.4 ± 0.3°	3.3 ± 0.1
G ratio	0.56 ± 0.01	0.55 ± 0.01	0.54 ± 0.01	0.56 ± 0.01°	0.55 ± 0.01	0.53 ± 0.01
Myelin sheath area (μm^2^)	8.9 ± 0.9	7.9 ± 0.51	13.9 ± 1.7^+^°	11.3 ± 0.9°	12.1 ± 1.3°	7.7 ± 0.6
Myelinated axon area (μm^2^)	5.2 ± 0.7	3.9 ± 0.4	6.9 ± 1.2°	6.4 ± 0.7°	7.5 ± 1.1°	3.5 ± 0.4
Myelinated axon diameter (μm)	2.3 ± 0.2	2.1 ± 0.1	2.6 ± 0.2°	2.5 ± 0.1°	2.5 ± 0.2°	1.8 ± 0.1
Unmyelinated axon area (μm^2^)	0.65 ± 0.07	0.62 ± 0.07	0.58 ± 0.03	0.57 ± 0.03	0.80 ± 0.10°^#^	0.56 ± 0.06
Unmyelinated axon diameter (μm)	0.76 ± 0.04	0.73 ± 0.03	0.73 ± 0.02	0.72 ± 0.02	0.83 ± 0.04°	0.68 ± 0.04

^+^Indicates difference compared to acute treated, °indicates difference compared to chronic control, ^#^indicates difference compared to chronic diabetic group.

For the acute groups, no differences were observer in all parameters evaluated. The comparison between the three chronic groups indicated that both treated and untreated diabetic groups showed larger average values for the myelinated fibers compared to the controls (One way ANOVA on Ranks, p < 0.001, for all parameters). Exception was made for the G ratio that was larger only on the diabetic group compared to control (One way ANOVA on Ranks, p < 0.001). The unmyelinated fiber area was larger on treated animals compared to both, diabetic and controls (One way ANOVA, p = 0.025; F = 4, DF = 2), while unmyelinated fiber diameter was larger on treated animals compared only to the controls (One way ANOVA, p = 0.033; F = 4, DF = 2).

The comparison between control groups, acute and chronic, showed larger myelinated fiber size for acute animals (p < 0.001 for all parameters). Exception is made for the G ratio that was not different. No differences for the unmyelinated fibers were found.

No differences were observed on the myelinated and unmyelinated fibers morphometric parameters between acute and chronic diabetic groups either treated or untreated.

**Figure 3:**
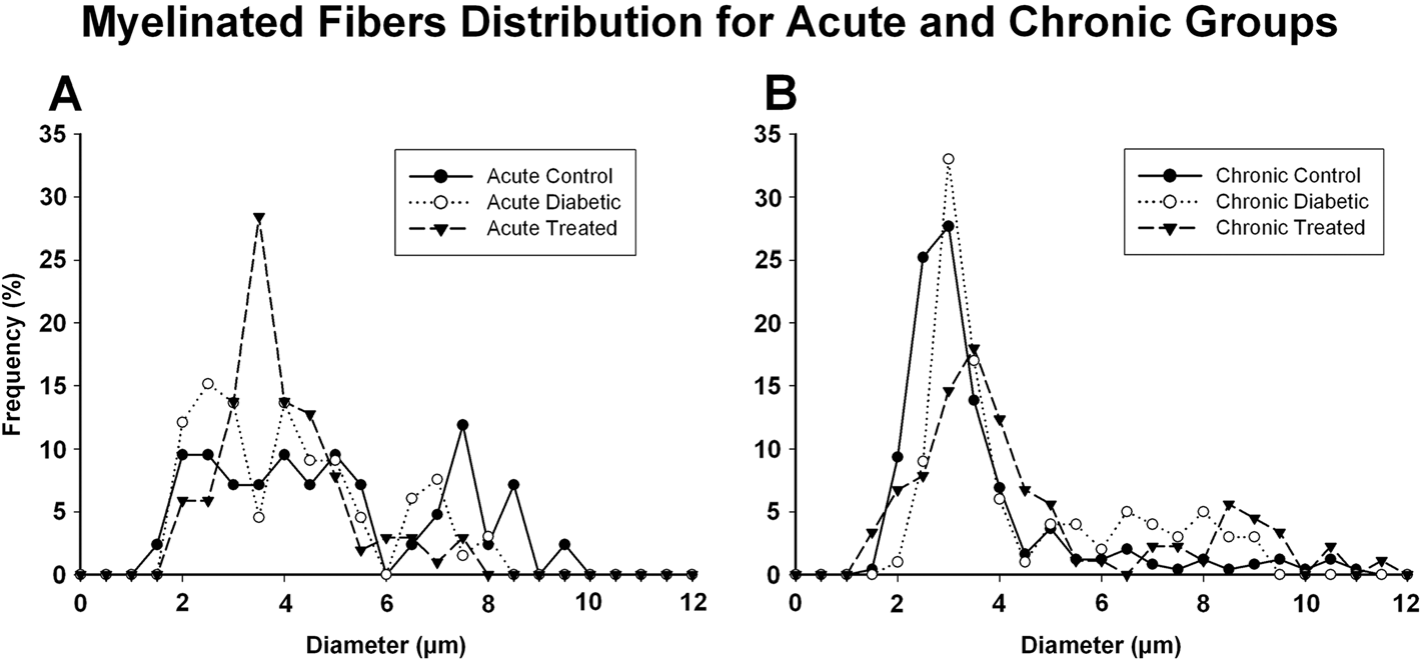
**Myelinated fibers diameter distributions on acute (A) and chronic (B) treated and untreated diabetic groups and respective controls.** All fibers from all nerves are represented. For all experimental groups, myelinated fibers distributions are bimodal in shape, with a clear point of separation between small and large fibers at 6.0 μm. The acute control group shows an homogeneous distribution of the small fibers while both diabetic groups, treated and untreated showed a larger percentage of small myelinated fibers, shifting the histograms to the left (One Way ANOVA on Ranks, p < 0.001). The chronic diabetic group also shows a shift of the distribution towards the small fibers, with a disorganization of the large fibers distribution (One Way ANOVA on Ranks, p < 0.001). The chronic treated group showed a reduced percentage of both small and large myelinated fibers (One Way ANOVA on Ranks, p < 0.001).

Myelinated fibers diameter distributions for acute and chronic groups are shown on Figure 3. Acute groups showed fibers diameters ranging from 1.5 to 10 μm, with a bimodal distribution: the small fibers group with diameters between 1.5 and 6.0 μm and the large fibers group with diameters between 6.0 and 10 μm. The acute control group showed an homogeneous distribution of the small fibers while both treated and untreated diabetic groups showed a larger percentage of small myelinated fibers, shifting the histograms to the left, confirmed by the observation of a higher percentage of large myelinated fibers on the control group (One Way ANOVA on Ranks, p < 0.001).

On the chronic groups, myelinated fibers diameters ranged from 1.5 to 12 μm, also with a bimodal distribution, very similar to that observed for the acute groups. The small fibers group showed diameters between 1.5 and 5.0 μm and the large fibers group with diameters between 5.0 and 10 μm. As for the acute diabetic, the chronic diabetic group also showed a shift of the distribution towards the small fibers, with a disorganization of the large fibers distribution (One Way ANOVA on Ranks, p < 0.001). The chronic treated group showed a reduced percentage of both small and large myelinated fibers (One Way ANOVA on Ranks, p < 0.001).

**Figure 4:**
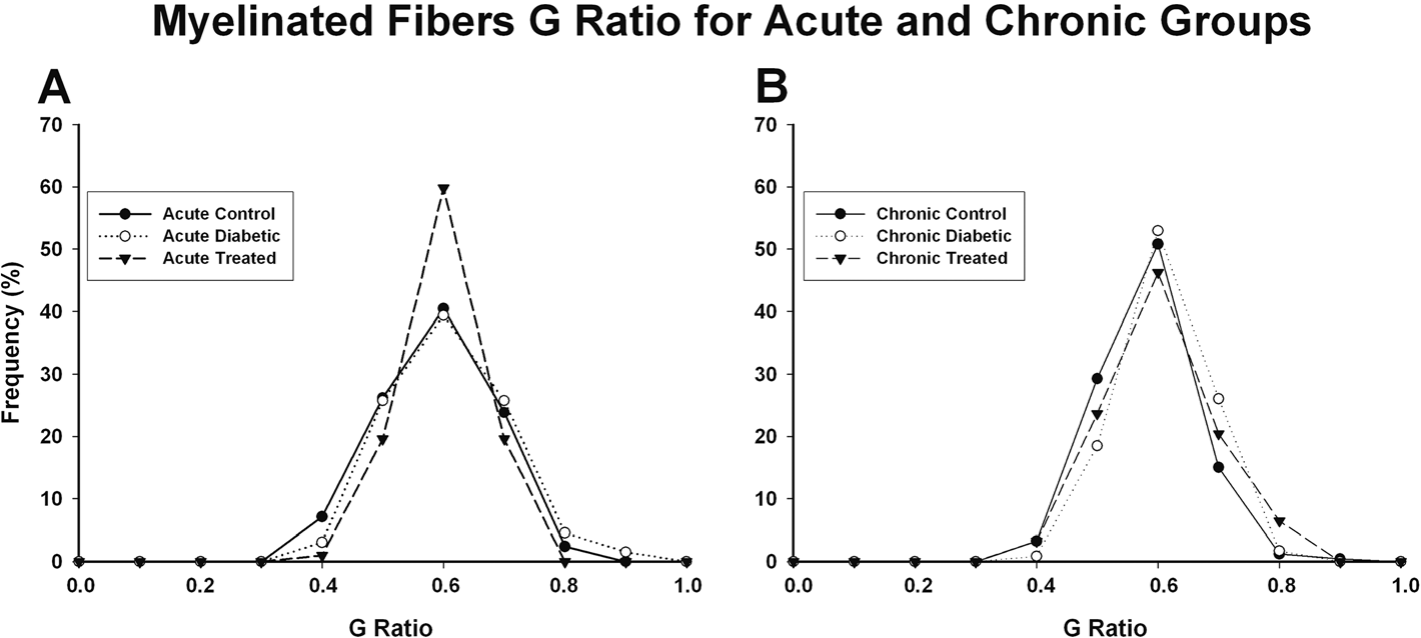
**Myelinated fibers G ratio on acute (A) and chronic (B) treated and untreated diabetic groups and respective controls.** All fibers from all nerves are represented. For all experimental groups the distributions are unimodal, with peaks at 0.6. Note that on acute diabetic group there is a slight shif of the distribution towards 1.0, suggestive of demyelination. This shift is more evident on the chronic diabetic groups, treated and untreated (One Way ANOVA on Ranks, p < 0.001).

Distributions of the myelinated fibers G ratio of all experimental groups are represented on Figure 4. For acute and chronic groups, regardless the experimental treatment, G ratio values ranged from 0.3 to 0.9, with unimodal distribution. The acute diabetic groups, treated and untreated, showed a slight shift of the histograms to the right (One Way ANOVA on Ranks, p < 0.001). This shift was more evident for the chronic treated and untreated diabetic groups, compared to controls.

**Figure 5:**
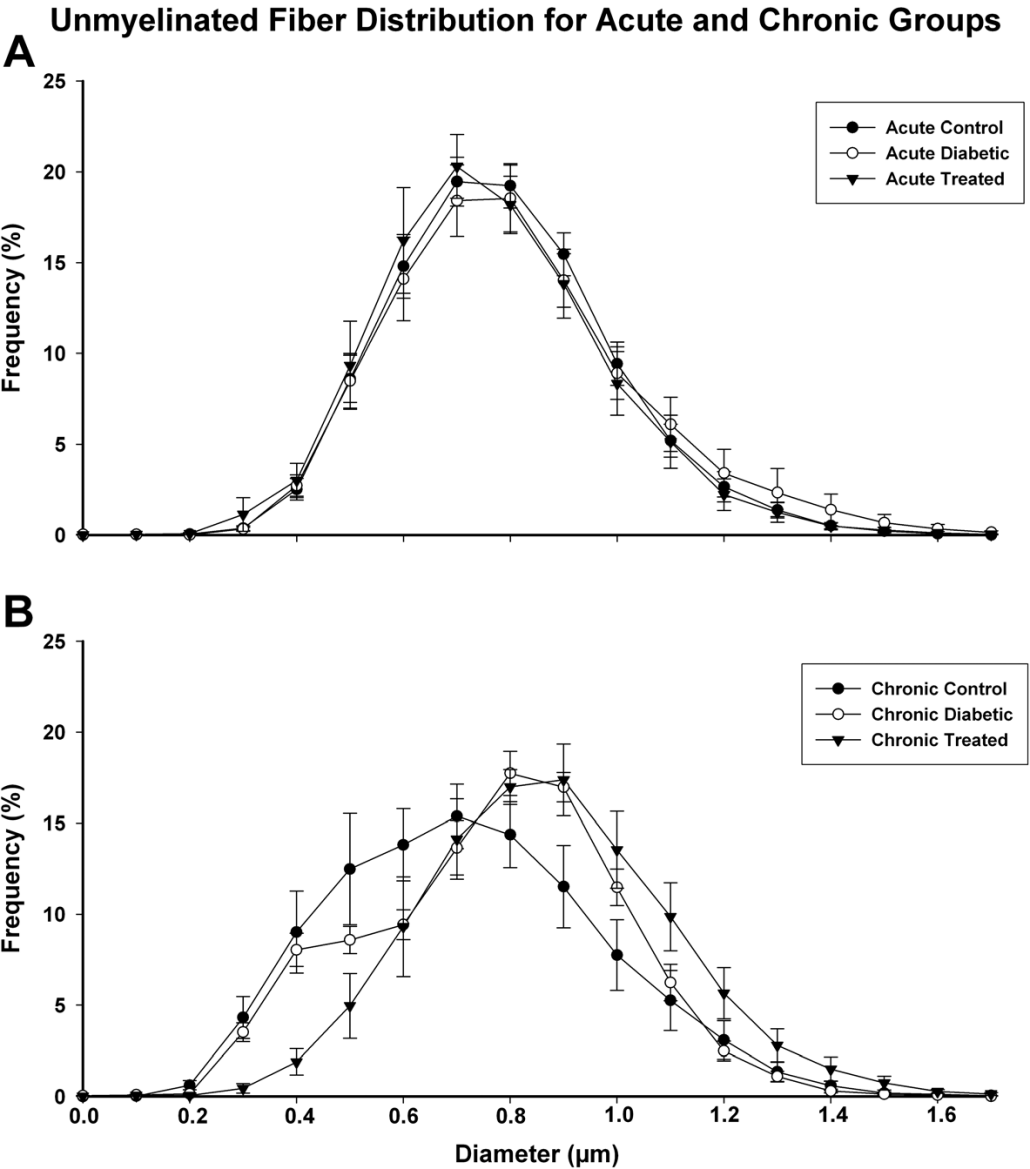
**Unmyelinated fiber distribution on acute (A) and chronic (B) treated and untreated diabetic groups and respective controls.** Average values are represented. The distributions are unimodal in all groups with the peak at 0.7 μm for acute groups and chronic control group. For the chronic diabetic groups, treated and untreated, there is a shift (One Way ANOVA on Ranks, p < 0.001) of the peak to 0.9 μm, indicating a loss of the small fibers.

Unmyelinated fibers diameters distributions are shown on Figure 5. For acute and chronic groups, regardless the experimental treatment, the values ranged from 0.1 to 1.7 μm, with unimodal distribution. For the acute groups, a sharp peak at 0.7 μm was observed, with a slight shift to the left on the treated group. On the chronic groups, the control group distribution was smooth, with a peak at 0.7 μm while the diabetic groups, treated and untreated, showed a clear shift to the right (One Way ANOVA on Ranks, p < 0.001), with peaks reaching 0.9 μm for both.

## Discussion

Our study show that the extrinsic renal nerve is affected by experimental diabetes, with the main morphological, ultrastructural findings, associated to the morphometric data, pointing to a small fiber loss. These alterations begin early, after 15 days of diabetes induction and are not prevented by conventional insulin treatment.

It is well known that the pathology of diabetic neuropathy is characterized by primary progressive nerve fiber loss [20,21] but somatic and automomic C fibers are not usually studied in the same region of the body [22] making the neuropathologic basis of the small fiber loss difficult to understand. Several investigators accessed the small fiber neuropathy in diabetes, focusing on the intraepidermal nerve fibers [23,24] but transmission electron microscopy studies are still scanty, particularly at early stages of diabetes. The extrinsic renal nerve provided an unique opportunity to investigate the involvement of small and large nerve fibers at once, since it has not only a large number of unmyelinated fibers, but also two distinct groups of myelinated fibers, as shown by the diameter distributions (Figure 4). Despite that advances on the pathogenesis of the glomerular lesions in diabetic nephropathy have been made, there is still a lot to understand. Our results suggest that lesions of extrinsic renal nerves might play a role on the glomerular functional alterations that occur very early in this diabetes model and these alterations could be involved in some degree to the development of the diabetic nephropathy.

More recently, altered function of mitochondria in metabolic diseases have been correlated to their aberrant morphology and growing evidence suggests that mitochondrial dynamics play an important role in diabetes establishment and progression [25]. Mitochondrial phenotype is abnormal in sensory neurons, Schwann cells and sympathetic ganglia in diabetes [26] but little information on nerve mitochondria is available. We show severe ultrastructural alterations on the mitochondria of the renal nerve axons from diabetic rats, as early as 15 days, and these alterations were delayed but not prevented by insulin treatment. An early work reveals that hyperglycemia in diabetes triggers nutrient excess in neurons, that mediates the phenotype changes in mitochondria, and the development of axon loss in sensory neuropathy is linked to this nutrient excess [26,27]. We are demonstrating, for the first time, the presence of mitochondrial damage on myelinated and unmyelinated fibers of the renal nerves in diabetes that might be contributing to the fiber loss observed in these nerves since early stages of the disease.

In somatic nerves, different types of sensations travel along fiber population with different average diameters. The renal nerves contain efferent post-ganglionic sympathetic fibers, thought to be exclusively unmyelinated. The myelinated fibers might be the afferent ones that would transmit sensory information from the kidneys to the central nervous system [19]. We suggest that the small myelinated fibers could be those afferent fibers related to baro- and chemoreflex, based on information from the aortic depressor nerve myelinated fibers sizes [28]. The chronic diabetic groups, treated and untreated, presented larger average values of myelinated fiber and axon area and diameter compared to controls. These data suggest a loss of thin myelinated fibers, shifting the average to higher values and also affecting the histogram distributions. A shift of the size distribution towards larger fibers observed in both, acute and chronic diabetic groups, associated with the shift to the right observed for the unmyelinated fiber size also in acute and chronic diabetic animals demonstrates thin fiber loss. This data is corroborated by the observation of smaller myelinated and unmyelinated fibers density in chronic diabetic animals, compared to controls, indicating the increasing severity of the fiber loss at chronic stages. This thin fiber loss could cause kidney denervation and also influence on the autonomic imbalance described in diabetes. Also, the shift observed on the unmyelinated fiber distribution, although less pronounced than the myelinated distributions, suggest the possible atrophy of thin axons in chronic diabetes, which might also contribute to the nephropathy, since the majority of the afferent renal nerves are unmyelinated. Patel and Zhang [29] observed that the volume reflex is reduced in STZ-induced diabetic rats after 15 days. They also showed that the natriuresis due to renal sympathoinhibion is blunted in response to volume expansion and the restoring the glucose levels to normal by insulin treatment in diabetic rats reversed the volume receptor reflex back to normal. We showed that insulin treatment delayed fiber loss in the short term diabetes but was not able to prevent it in long term. Yagihashi and Sima [30] discussed that the characteristic of the autonomic neuropathy involving both sympathetic and parasympathetic peripheral nerves is a structural change, consisting in dystrophic axonal alterations. These authors [30] affirm that the important morphometric changes such as fiber atrophy and loss of unmyelinated fibers was progressive during the course of diabetes, which is compatible with the results from the present study.

Despite the fact that the literature describes preferably a reduction of the myelinated fiber and axon sizes in somatic nerves of STZ-induced diabetic rats [31-33] our results show, in an essentially autonomic nerve, a small myelinated fibers loss, accompanied by degeneration and atrophy of the unmyelinated fibers, in this model of diabetes. This is suggestive that somatic and autonomic nerves react in distinct ways to diabetes. Another interpretation is because somatic nerves have a large number of myelinated fibers, the small fiber loss observed in autonomic nerves, might not be easily or readily identified in somatic ones, particularly because most studies used light microscopy, that has no resolution for the unmyelinated fibers investigation.

It is well known that Schwann cells are important in the regeneration processes that follow nerve injury. Nerves that are experiencing regeneration will have a larger number of Schwann cells due to their augmented duplication rate under these circumstances [34]. We showed that density of Schwann cell is larger in chronic diabetes, treated and untreated, indicating possible regeneration of the large myelinated fibers. In fact, this result, together with the larger average G ratio in chronic diabetes and the shift to the right on the G ratio distribution, is a morphological indication that the remaining myelinated fibers on the chronic diabetic nerves have thinner myelin sheath, compatible with multiplication of the Schwann cells in order to accomplish the regeneration process. Regeneration of fibers is a slow process and this is probably the reason that these alterations were not present in acute diabetic animals.

There has been increasing evidence that microvascular pathological abnormality and ischemia may be involved in the pathogenesis of diabetic neuropathy [20]. In fact, Fazan et al. [7] demonstrated severe damage of the endoneural vessels present on both STZ groups, besides the insulin treatment, using a similar chronic diabetes model from this study. We showed that at early stages of diabetes, vascular damage occurs in the endoneural space of the renal nerves that increase in severity on chronic diabetes regardless insulin treatment.

It is important to mention that the insulin treatment is demonstrated to prevent or correct the axonal atrophy caused by STZ-diabetes in the large myelinated fibers [6,35-37] as well as the axonal diameter and their distributions [33]. The STZ-diabetes model is widely used to investigate experimental diabetic peripheral neuropathies [6,36,38,39], but few studies have performed a detailed assessment of either unmyelinated fibers or capillary morphology in this animal model, particularly on renal nerves. Thus, the present study adds useful information for further investigations on the ultrastructural basis of nerve function in diabetes and also provides support for the role of the renal nerve neuropathy on the development of the diabetic nephropathy.

## Conclusions

The experimental diabetes, induced by a single intravenous injection of STZ, in adult male Wistar rats, caused small fiber loss in the renal nerves, probably due to the early mitochondrial damage. Conventional treatment with insulin was able to correct the weight gain and metabolic changes in diabetic animals, without, however, correcting and / or preventing damage to the thin fibers caused by STZ-induced diabetes. The kidney innervation is impaired in this diabetic model suggesting that alterations of the renal nerves may play a role in the development of the diabetic nephropathy.

## Methods

Experiments were performed on male adult Wistar rats, born and raised in a carefully regulated environment maintained at 21°C - 23°C, 40% - 70% relative air humidity, and 12/12 hr light/dark cycle, receiving tap water and normal rat chow *ad libitum*, throughout the experiment. Six experimental groups were used (N = 10 for each group): 1) Rats 15 days after STZ injection, without insulin treatment (acute diabetic group); 2) Rats 15 days after STZ injection, treated with insulin (acute treated group); 3) Control animals for acute groups, which received only vehicle (citrate buffer) 15 days before the experiments (acute control group); 4) Rats 12 weeks after STZ injection, without insulin treatment (chronic diabetic group); 5) Rats 12 weeks after STZ injection, treated with insulin (chronic treated group) and 6) Control animals for chronic groups, which received only vehicle (citrate buffer) 12 weeks before the experiment (chronic control group).

STZ (60 mg/Kg) or citrate buffer injections as well as insulin treatment (9 IU/Kg on a daily basis, at 7:00 PM) were performed as described previously [7,33,40]. The animals were considered diabetic when the blood glucose levels were higher than 350 mg/dl. On the STZ injected animals, the onset of diabetes occurred rapidly and was identified by polydipsia and polyuria. Non-fasting blood glucose (mg/dl) was determined with a glucose analyzer (Beckman Instruments, Inc., Brea, CA, USA) 3 days after STZ injection and immediately before the experiments, in blood droplets collected from an incision at the tip of the tail.

On the final experimental day (15 days or 12 weeks after STZ injection), animals were anesthetized with sodic thiopentahl (40 mg/kg, i.p.) and a polyethylene catheter was inserted into the right carotid artery and connected to a pressure transducer (Statham PB23Gb) for direct measurement of arterial pressure (AP) and heart rate (HR). Left extrinsic renal nerves (the major branch from the aortic-renal plexus entering the kidney) were exposed and their activity was recorded simultaneously with AP as previously described [19]. The characteristic recording of the sympathetic nerve activity during the diastolic phase of the cardiac cycle was obtained for all nerves studied.

All procedures adhered to *“The ARRIVE guidelines: Animal Research: Reporting In Vivo Experiments, originally published in PLoS Biology, June 2010”* and were approved by the Institutional Ethics Committee for Animal Research (CETEA–Comitê de Ética em Experimentacão Animal, protocol number 001/2004). A conscious effort was done to minimize the number of animals used.

### Fascicles and myelinated fibers study

The methods for identification, dissection and histological preparation of the nerves were described elsewhere [18,19]. Before embedding in epoxy resin (EMbed 812®, Electron Microscopy Sciences, Hatfield, PA, USA), the nerves were oriented to permit semi-thin (0.5 to 1.0 μm thick) transverse sections of the fascicles, which were stained with 1% toluidine blue and observed under the oil immersion lens of an Axiophot II photo-microscope (Carl Zeiss, Jena, Germany). The light microscopy study was carried out for the fascicle morphometry and also in order to check the quality of the histological preparation of the nerves to be studied under transmission electron microscopy. The fascicle images were transmitted via a digital camera (TK-1270, JVC, Victor Company of Japan, Ltd., Tokyo, Japan) to an IBM PC where they were digitized. With the aid of an image analysis software (KS 400, Kontron 2.0, EchingBeiMüchen, Germany) the total number of myelinated fibers present in each fascicle were identified by visual inspection and counted. The area and diameter of the nerve fascicles (excluding the perineurium), as well as the area and diameter of the myelinated fibers and respective axons present in the endoneural space were measured and the myelinated fiber density was calculated. The ratio between the myelinated axon and respective myelinated fiber diameters, G ratio (a measure of degree of myelination), was obtained [41,42] and the myelin sheath area was calculated for each myelinated fiber measured. Histograms of the population distribution of the myelinated fibers and respective axons, separated into class intervals increasing by 0.5 μm were constructed. Histograms of the G ratio distribution separated into class intervals increasing by 0.1 were also constructed.

### Unmyelinated fibers study

For the transmission electron microscopy studies, thin transverse sections were mounted on 2 × 1 slot grids, covered with formvar 0.5% solution, stained with lead citrate and uranyl acetate, and observed under the transmission electron microscope (JEM-1230, JEOL-USA, Inc., Peabody, MA, USA), equipped with a digital camera. The endoneural space of the nerves was fully scanned and, at least 15 digital images were obtained for each nerve, without overlap of the microscopic fields. Using the image analysis software (KS 400), the unmyelinated fibers were counted and their area and diameter were measured. The density of the unmyelinated fiber was calculated and the ratio between unmyelinated/myelinated fibers was determined, as also the percentage of the fascicular area occupied by them (percentage of occupancy). The number of Schwann cell nuclei present in each transverse section of the renal nerves was counted and their density calculated. Histograms of the unmyelinated fiber population were constructed and separated into class intervals increasing by 0.1 μm.

### Statistical analysis

All data are presented as mean ± standard error of mean (SEM). The Kolmogorov-Smirnov test was applied to verify the normal distribution for weight, biochemical and morphometric data, using Sigma Stat software, version 3.01 (Jandel Scientific), followed by the Levene test of medians for variance equivalence.

Body weight, mean arterial pressure (MAP) and heart rate (HR) data were compared between the 3^rd^ day after injection and the final experimental day by the paired Student’s *t*-test and among groups by the one-way ANOVA, followed by Holm-Sidak post-test, provided that these data showed normal distribution. Blood glucose data did not show normal distribution and were compared between the 3^rd^ day of injection and the final experimental day by the non-parametric test of Wilcoxon for paired samples, and among groups by the analysis of variance on Ranks, followed by Dunn’s post-test.

Morphometric data that passed the normality and variance equivalence tests were compared by unpaired Student’s t-test (between acute and chronic groups) and one way ANOVA followed by a Holm-Sidak post-test between groups in the same experimental time. Data that did not show normal distribution were compared using the non-parametric test of Mann–Whitney between acute and chronic groups, while groups in the same experimental time were compared by the analysis of variance on ranks, followed by Dunn’s post-test.

Comparisons between histograms were made by one-way analysis of variance (ANOVA) on Ranks, provided that all distributions did not pass on the normality test. All differences were considered significant when p < 0.05.

## Authors’ contributions

KLS was responsible for acquisition, analysis and interpretation of data and drafting the manuscript; LSS was responsible for acquisition and analysis and drafting the manuscript; RSF was responsible for acquisition of data and analysis; MCDBBOM was responsible for acquisition of data and analysis; JAC carried out the renal nerve isolation and recording; HCS trained the student on acquisition of physiological data and analysis; RAN trained the students on the acquisition of transmission electron microscopy data and analysis and VPSF was responsible for conception and design, training the student on light microscopy data acquisition, interpretation of all data (physiological and morphological), revising the manuscript critically and for given final approval of the version to be published.
